# A psychometric study of the team psychological safety scale and sport psychological safety inventory in Swedish elite sports

**DOI:** 10.1038/s41598-025-06963-1

**Published:** 2025-06-20

**Authors:** Carolina Lundqvist, Stéphane Bermon, Toomas Timpka

**Affiliations:** 1 Department of Behavioural Sciences and Learning, Linköping University, Linköping, SE-581 83 Sweden; 2 Athletics Research Center, Linköping University, Linköping, Sweden; 3 Health and Science Department, World Athletics, Monaco, Monaco; 4 LAMHESS, Université Côte d’Azur, Nice, France; 5 Regional Executive Office, Region Östergötland, Linköping, Sweden

**Keywords:** Athletics, Elite athlete, Mental health, Measurement, Psychological safety, Orienteering, Medical research, Psychology

## Abstract

Studies investigating psychological safety in sports and non-sports contexts have mostly utilized the universal Team Psychological Safety Scale (TPSS) aimed for performance development in professional teams. The Sport Psychological Safety Inventory (SPSI) has recently been introduced for psychological safety measurement specifically in sports. The aim of this study was to compare the psychometric properties of the TPSS and the SPSI within an elite sport context. A cross-sectional survey was used to collect data for assessment of the internal consistency, factorial validity, construct validity and measurement invariance of the TPSS and the SPSI. Complete data sets were provided by 371 elite Athletics athletes (track and field) and orienteers. Both the TPSS (ω = 0.72) and the SPSI subscales (range: ω = 0.81-0.88) showed acceptable internal consistency. Confirmatory factor analyses indicated a mediocre to good model fit for the TPSS and the SPSI three-factor correlated structure. The TPSS and the SPSI subscale ‘mentally healthy environment’ showed a moderate correlation. Measurement invariance tests suggested the TPSS to be fully invariant across genders, while the SPSI was found non-invariant. The study shows that the TPSS appears sound for assessing psychological safety in elite sports, while caution is needed when using the SPSI.

## Introduction

Building on research initially rooted in organizational psychology that has demonstrated quantifiable associations between levels of psychological safety and team performance under pressure, as well as wellbeing and job satisfaction in individual team members^1–4^, a growing body of literature discusses the application of the concept in sports settings^5,6^. An increasing number of studies suggest that psychological safety is associated with adaptive outcomes in sports, for example, a good quality in the coach-athlete relationship, resilience, and mental health^7–9^. Most studies investigating psychological safety have utilized Edmondson’s Team Psychological Safety Scale (TPSS)^3,10^ aimed at performance development in professional teams. According to the operational definition of the TPSS, psychological safety is *‘a shared belief that the team is safe for interpersonal risk taking’*[^10^, p. 354]. This definition characterizes psychological safety as mutual respect and trust among team members, where speaking up or being oneself does not lead to negative consequences. The emphasis is on open communication without fear of embarrassment or punishment, which is also linked to factors like leadership and organizational policies^10–13^.

In 2021, the International Olympic Committee (IOC) defined psychological safety in sports as ‘*the creation of an athletic environment where athletes feel comfortable being themselves*,* can take necessary interpersonal risks*,* have the knowledge and understanding of mental health symptoms and disorders*,* and feel supported and comfortable in seeking help if needed*’[^14^, p. 34]. This definition, in addition to interpersonal coherence and the aspect of open communication, encompasses the individual’s understanding of mental health and readiness for help-seeking. Given the contextual differences between organizational (e.g., business, healthcare) and sports settings, as well as the semantic gap in the interpretation of psychological safety across the contexts^6^, it is unsurprising that a systematic review of the literature on psychological safety in sports reported that only 30% (*n* = 67) of articles investigating this concept provided a clear definition^5^. The term psychological safety was in sports often used as a broad label to describe phenomena ranging from threat and harm to general impressions of inclusivity, equality, and respect. Based on their review, Vella and colleagues proposed defining psychological safety in sports as *‘the perception that one is protected from*,* or unlikely to be at risk of*,* psychological harm in sport’*[^5^, p. 15].

One of the few instruments adapted for the measurement of psychological safety in sports is the Sport Psychological Safety Inventory (SPSI), which includes three subscales: Mentally healthy environment, mental health literacy, and low self-stigma^15^. The initial validation study conducted among Australian elite athletes and coaches supported a three-factor correlated structure. Low scores on the mentally healthy environment subscale and high scores on the low self-stigma subscale were associated with moderate mental health distress caseness, but scores on the mental health literacy subscale were not predictive of such distress^15^.

Although both the TPSS and SPSI have been developed to measure the concept of psychological safety and both scales have been applied in sports^7–9,15,16^, they diverge in their operational definitions. It remains unclear how these scales conceptually relate to each other and to important endpoints (e.g., health, coach-athlete relationship, performance) in sports, which has implications for the interpretation and conclusions of studies. The data collection for the SPSI validation was in addition performed during the early stages of the COVID-19 pandemic, a period characterized by exposure to strong, yet transient, psychological stressors. The authors therefore called for further validation of the psychometric properties of the inventory, as well as replication studies in diverse samples and cross-cultural settings^15^. Responding to the call for further analyses, the aim of this study was to investigate the psychometric properties, including internal consistency, factorial validity, construct validity and measurement invariance, of the TPSS and the SPSI in a Swedish elite sport context.

## Methods

### Participants

Swedish Athletics (track and field) athletes and orienteers, ranging from junior national sub-elite to senior international elite categories and aged ≥ 15 years, were invited to participate. A total of 371 athletes (Athletics: *n* = 233, females = 125; orienteering: *n* = 138, females = 73) completed the questionnaire. The mean ages were 18.72 years (SD = 4.73) for the Athletics sample and 18.93 (SD = 3.90) for the orienteering sample. Table 1 presents descriptive statistics for participants’ mean age, the age at which they began training in their sport, training hours per week, and the number of coaches they were currently trained by, categorized by competitive levels. The diverse group provided a comprehensive representation of the athletic spectrum within these sports.

**Table 1:** Descriptives of participants competitive levels, age, age when they started training the sport, training hours/week and number of coaches.

Variable	Age			Age when started training the sport			Training hours/week			Number of coaches		
	Females	Males	Total	Females	Males	Total	Females	Males	Total	Females	Males	Total
	M (SD)	M (SD)	M (SD)	M (SD)	M (SD)	M (SD)	M (SD)	M (SD)	M (SD)	M (SD)	M (SD)	M (SD)
*Athletics* (n = Females/Males/Total)												
National junior elite (*n* = 21/13/34)	17.38 (2.01)	20.08 (8.21)	18.41 (8.74)	8.00 (2.30)	9.92 (4.42)	8.74 (3.35)	8.24 (3.67)	12.31 (10.18)	9.79 (7.06)	2.90 (1.04)	3.46 (1.61)	3.12 (1.30)
International junior elite (*n* = 77/51/128)	17.22 (1.41)	17.78 (5.08)	17.45 (3.38)	8.06 (2.49)	9.08 (2.63)	8.47 (2.58)	11.35 (3.86)	10.76 (4.14)	11.12 (3.97)	3.08 (1.33)	3.20 (1.18)	3.13 (1.27)
National senior elite (*n* = 15/20/35)	17.33 (1.76)	17.25 (1.12)	17.29 (1.40)	7.73 (2.12)	9.35 (3.57)	8.66 (3.10)	11.93 (3.95)	14.75 (6.16)	13.54 (5.45)	3.00 (1.36)	3.70 (1.38)	3.40 (1.40)
International senior elite (*n* = 10/19/29)	23.20 (3.58)	25.63 (6.84)	24.79 (5.97)	10.70 (5.33)	9.42 (2.61)	9.86 (3.73)	12.60 (6.19)	12.63 (4.27)	12.62 (4.90)	2.50 (0.71)	2.84 (1.01)	2.72 (0.92)
Other level/below elite (*n* = 2/5/7)	23.50 (2.12)	26.40 (2.79)	25.57 (2.82)	10.00 (2.83)	11.60 (5.37)	11.14 (4.60)	16.00 (5.66)	13.40 (7.13)	14.14 (6.39)	2.50 (0.71)	2.20 (0.45)	2.29 (0.49)
Total sample (*n* = 125/108/233)	17.84 (2.52)	19.74 (6.27)	18.72 (4.73)	8.26 (2.81)	9.41 (3.20)	8.79 (3.04)	11.07 (4.27)	12.14 (5.80)	11.57 (5.05)	2.98 (1.24)	3.21 (1.26)	3.09 (1.25)
*Orienteering* (n = Females/Males/Total)												
National elite (juniors) (*n* = 16/12/28)	17.44 (2.19)	17.67 (2.74)	17.54 (2.40)	6.44 (3.44)	7.92 (4.93)	7.07 (4.13)	3.81 (2.19)	3.50 (1.88)	3.68 (2.04)	4.44 (1.59)	4.17 (1.19)	4.32 (1.42)
International elite (junior) (*n* = 43/32/75)	17.33 (1.02)	17.53 (0.84)	17.41 (0.95)	5.56 (4.02)	6.13 (3.87)	5.80 (3.94)	3.72 (1.99)	3.48 (2.17)	3.62 (2.06)	4.53 (1.12)	4.78 (1.18)	4.64 (1.15)
National elite (senior) (*n* = 4/2/7)	18.00 (1.22)	16.50 (0.71)	17.57 (1.27)	7.60 (3.21)	8.50 (4.95)	7.86 (3.34)	6.60 (2.30)	5.00 (2.83)	6.14 (2.34)	3.60 (1.67)	4.00 (0.00)	3.71 (1.38)
International elite (senior) (*n* = 3/11/14)	23.00 (4.36)	22.27 (3.23)	22.43 (3.32)	4.33 (4.51)	6.27 (3.95)	5.86 (3.98)	4.67 (3.79)	6.40 (4.79)	6.00 (4.49)	2.67 (1.53)	2.18 (1.40)	2.29 (1.38)
Other level (below elite) (*n* = 6/8/14)	28.83 (4.53)	25.75 (5.78)	27.07 (5.33)	5.67 (4.50)	3.88 (3.48)	4.64 (3.89)	11.17 (7.52)	6.43 (4.20)	8.62 (6.20)	2.33 (1.37)	3.38 (2.07)	2.93 (1.82)
Total sample (*n* = 73/63/138)	18.58 (3.80)	19.34 (3.99)	18.93 (3.90)	5.85 (3.86)	6.28 (4.12)	6.05 (3.97)	4.59 (3.52)	4.34 (3.15)	4.47 (3.35)	4.19 (1.45)	4.03 (1.62)	4.12 (1.53)

### Study design and data collection

This study employed a cross-sectional design by using an online survey. The data utilized in this study are part of a larger data collection, which included standardized questionnaires related to mental health, psychological safety, and other environmental or health prerequisites in elite Athletics and orienteering. With support from the Swedish Athletics Federation and the Swedish Orienteering Federation, an invitation containing a QR code and a weblink to the survey was distributed to all National Sports High Schools, elite clubs, high-performance environments, and national teams within the respective federations. Data were collected from April 2023 to March 2024, and the survey was completed anonymously. Data collection for Athletics was conducted using the Lynes platform (lynes.io), while data collection for orienteering was conducted using the Artologik Survey&Report platform (artologik.com). The same survey was administered on both platforms. The transition of platform was driven by technical considerations and was not considered to impact on the quality of data collection.

### Measures

Demographics collected included age, age when the participants had started training the sport, self-assigned gender, number of training hours/week and number of coaches they currently were trained by.

The Team Psychological Safety Scale (TPSS)^10^ consists of seven items and was back-translated from English to Swedish. Originally developed for use in organizations, the scale assesses team psychological safety, such as the extent to which team members feel safe taking interpersonal risks like admitting mistakes or asking for help. Respondents rate each item on a 7-point scale, ranging from 1 (“strongly disagree”) to 7 (“strongly agree”). Three items (item 1,3,5) are reverse scored. Total scores range from 7 to 49, with higher scores indicating greater perceived psychological safety. While support for the reliability and validity of the TPSS has been reported both in non-sports and sports contexts^9,10,16^, one study found potential problems related to item 6 when the scale was used in sports^7^.

The eleven-item Sport Psychological Safety Inventory (SPSI)^15^ was back-translated from English to Swedish. The SPSI operationalizes psychological safety into three subscales: mentally healthy environment (four items), mental health literacy (four items), and low self-stigma (three items). Respondents rate their answers on a five-point scale ranging from 0 (“strongly disagree”) to 4 (“strongly agree”). Three items (item 9,10,11) are reverse scored. Higher scores on the subscales indicate higher levels of perceived mentally healthy environment (total score range: 0–16), mental health literacy (total score range: 0–16), and lower self-stigma (total score range: 0–12). The initial validation provided support for the scale’s internal consistency and a three-factor correlated structure^15^.

A Swedish version of the fourteen-item Hospital Anxiety and Depression Scale (HADS) was utilized to assess anxiety (seven items) and depression (seven items)^17,18^. Responses are scored on a four-point scale (ranging from 0 to 3), with total scores for each subscale ranging from 0 to 21. Higher scores indicate greater levels of anxiety and depression symptoms, with a cut-off score of ≥ 11 recommended to identify probable cases of clinically significant anxiety or depression disorders^19^. The HADS is widely used in Swedish healthcare and has been extensively validated, demonstrating good psychometric properties^17–21^.

A Swedish version of the 11-item Coach-Athlete Relationship Questionnaire (CART-Q)^22,23^ was used to assess the coach-athlete relationship in terms of commitment, closeness, and complementarity. Respondents rated their responses on a seven-point scale ranging from 1 (“strongly disagree”) to 7 (“strongly agree”). The scale has been validated in various languages, demonstrating adequate psychometric properties^22,23^. In the present study, linguistic problems with the Swedish wording of item 2 (“I feel committed to my coach”) were identified during data screening, resulting in an unacceptable low McDonald’s omega. Problems with this item in the Swedish version of the CART-Q has also previously been identified^23,24^. Consequently, this item was removed in this study, while the remaining ten items were retained. The total scores for the 10-item version of the CART-Q in this study ranged from 10 to 70. A high score indicates a good quality coach-athlete relationship.

### Statistical analyses

The sample characteristics, including means and standard deviations (SD), were analyzed using descriptive statistics, and scale reliability was calculated using McDonald’s omega (ω). Mann–Whitney U tests were conducted to explore differences between sports (Athletics athletes and orienteers) as well as between female and male athletes. Effect size for the Mann–Whitney U test (r) was calculated, with < 0.3 representing a small effect, and thresholds for medium and large effects being 0.3 and 0.5, respectively^25^. To evaluate the construct validity of the TPSS and SPSI, Spearman rank-order correlations were calculated with scores from instruments measuring the coach-athlete relationship (CART-Q) and mental health (HADS for anxiety and depression). Given that psychological safety as a construct has been suggested to be associated with a higher quality in the coach-athlete relationship and favorable conditions to support athletes’ mental health^7–9^, we hypothesized psychological safety scores on both scales to be positively related to CART-Q scores and negatively related to HADS scores. Both the Mann-Whitney U test and Spearman rank-order correlation are non-parametric tests appropriate for the ordinal data that were used in this study. None of the tests assume normal distribution of data, as they are based on ranks of scores. However, the Mann-Whitney U test assumes similar distribution shapes across independent groups, while the Spearman rank-order correlation assumes independent observations between pairs of variables^25^. Descriptive analyses and non-parametric tests were performed using SPSS Statistical Package version 29.

Confirmatory factor analyses (CFA) were conducted using MPlus version 8.8 to validate the factor structure of the measurement models for the TPSS and SPSI. Before conducting the CFA analyses, tolerance and variance inflation factor (VIF) was investigated to diagnose collinearity. Multicollinearity is indicated by a VIF above 4 or tolerance below 0.25 and no indication of collinearity was found in the data. Additionally, Mahalanobis distance was explored to detect multivariate outliers. The Mahalanobis distance measures the distance of a case from the centroid of the other cases, with the centroid being the point where the means of all variables intersect. A case is considered a multivariate outlier if it meets the chi-square (χ²) criterion with degrees of freedom and a significance level of *p* >.001^26^. To prevent multivariate outliers from disproportionately influencing the results and distorting the overall model fit, which could lead to misleading conclusions about the model’s adequacy, multivariate outliers were removed prior to conducting the CFA: s. Missing data were handled using pairwise deletion.

**Fig. 1:**
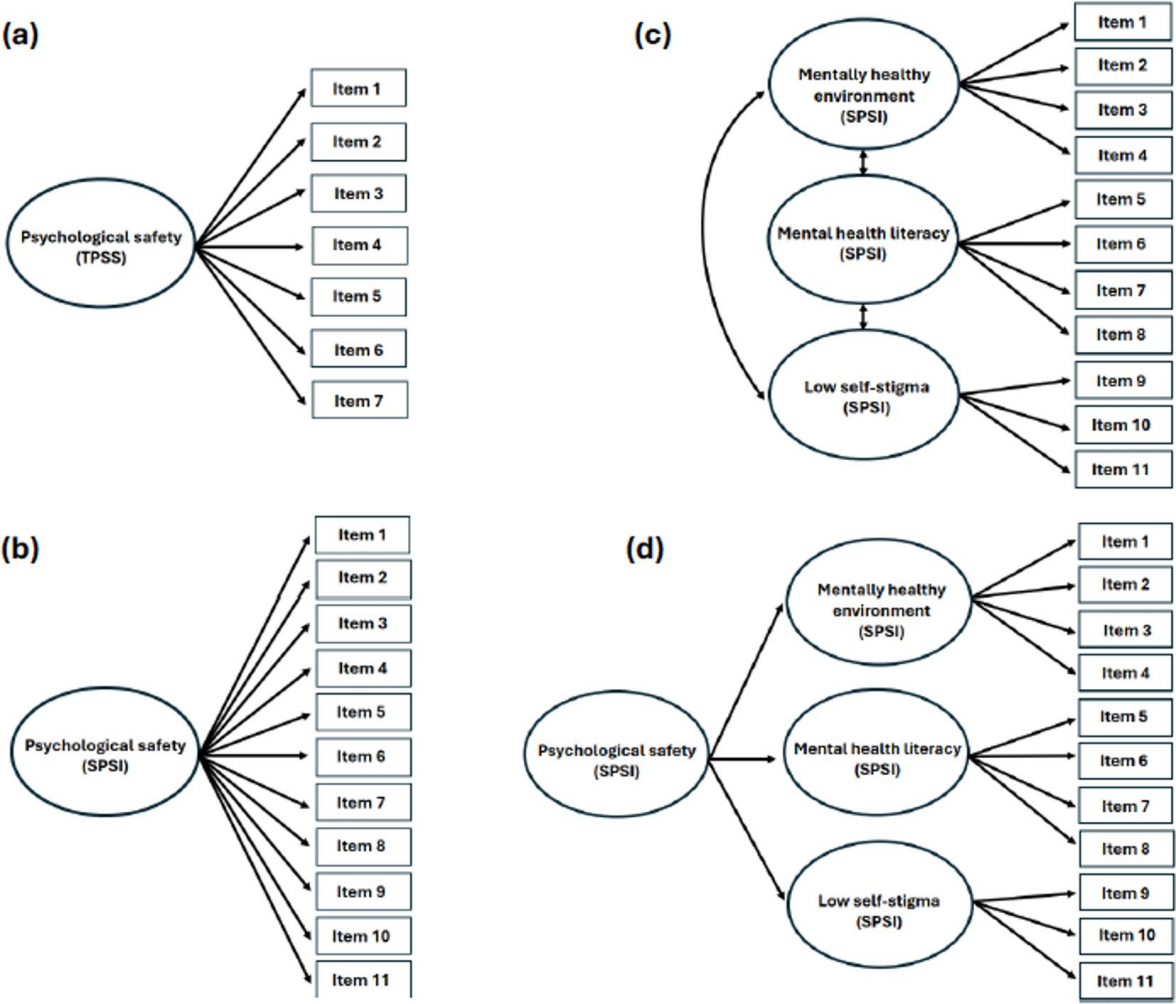
Á priori hypothesized measurement models tested for the TPSS (a) and the SPSI (b-d).

The four á priori hypothesized measurement models tested are displayed in Fig. 1. For the TPSS, and based on Edmundson’s original scale^10^, a one-factor hypothesized á priori measurement model was tested (Fig. 1a).

For the SPSI, three hypothesized á priori measurement models were tested based on findings in the initial validation study conducted by Rice et al.^15^:

1. A first order measurement model with one latent factor (Fig. 1b).
2. A first order measurement model with three latent correlated factors (mentally healthy environment, mental health literacy, low self-stigma (Fig. 1c).
3. A higher order measurement model with one higher order factor (psychological safety) and three latent factors (mentally healthy environment, mental health literacy, low self-stigma) (Fig. 1d).

**Fig. 2:**
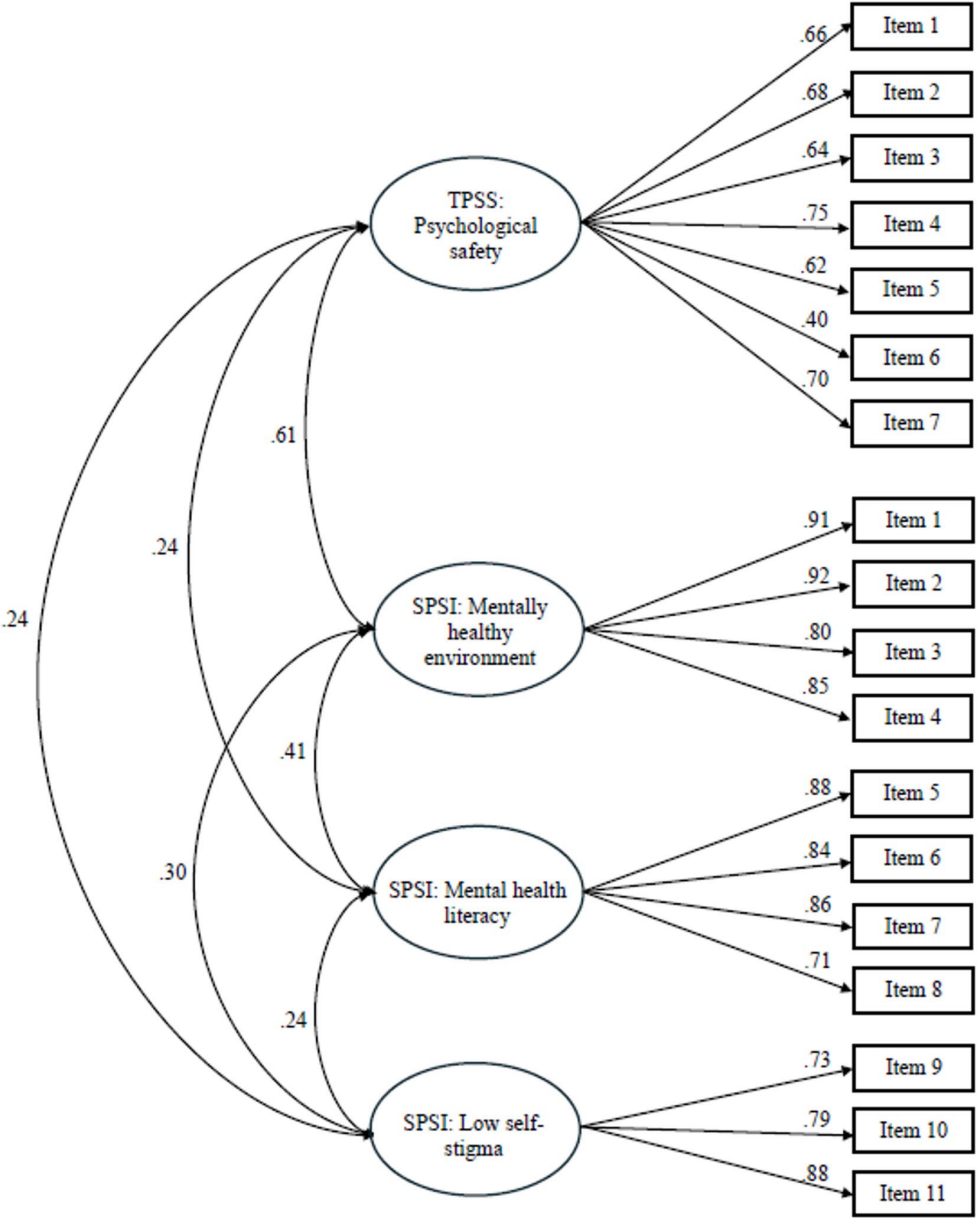
Post hoc confirmatory factor analysis with the one latent factor measurement model of the TPSS and the three-factor correlated measurement model of the SPSI analysed in one model. Standardized correlations between the TPSS and the SPSI latent variables and standardized factor loadings.

To examine the interrelationship between the latent factors in the TPSS and the SPSI, a post hoc analysis was performed to analyze the scales together. The measurement models that displayed the most acceptable model fit for each scale were combined into a comprehensive model, with the latent factors from the two scales specified as correlated (see Fig. 2).

To assess the model fit of the hypothesized models, and because ordinal data was used, weighted least squares mean and variance (WLSMV) estimation was adopted to provide robust parameter estimates and standard errors^27^. Model fit was evaluated using the comparative fit index (CFI) and the root mean square error of approximation (RMSEA)^28^. A good model fit is indicated by CFI > 0.95 and RMSEA < 0.06. For RMSEA, values between 0.08 and 0.10 indicate a mediocre fit while values > 0.10 indicate a poor fitting model^28–30^.

Measurement invariance was tested to evaluate the equivalence of the scales (TPSS and SPSI) across gender. Measurement invariance evaluates if a construct is interpreted and assessed similarly across groups and is a prerequisite for group mean comparisons^31,32^. This involves analyses of increasingly constrained and nested models. First, configural invariance is established by analyzing the model fit achieved with only the factorial structure constrained across the groups of females and males. This step assesses the invariance of the dimensional model’s configuration across both groups, also serving as the baseline for further steps in the measurement invariance tests. In the second step, metric invariance is tested by constraining the factor loadings across gender. The third step focusses on scalar invariance where the item thresholds are required to be identical for both genders. We adopted the MPlus shortcut option that automatically runs multiple group models to test measurement invariance, using the settings configural, metric and scalar^33^. To compare if subsequently more constrained models are significantly different (*p* <.05) and thereby not invariant, the shortcut option provides chi-square difference testing with scalar corrections for WLSMV^33–36^. The chi-square difference test is an exact fit approach, but a limitation is that the test can be overly sensitive particularly when using large samples^37^. Indices of approximate fit have been discussed as a solution, usually by calculating CFI (∆CFI) or RMSEA (∆RMSEA) differences^32,37,38^. However, these indices are descriptive. There is no clear consensus on which fit indices and cut-offs should be used to assess misspecification under various conditions^32,37,38^. For example, simulation analyses show that ∆CFI may retain both well-fitting and poor-fitting models, imposing uncertainty regarding the appropriate cut-off^32^. The ∆RMSEA has been reported to lack sensitivity and could therefore potentially mask misfit, particularly for models with large initial degrees of freedom^37^. While additional indices are proposed (e.g., RMSEA_D_) they have also met objections^37,39^. A discrepancy between modification index values and chi-square difference tests in MPlus can also be observed when using WLSMV for ordinal data. This is due to the adjustments made in the chi-square difference test to accommodate this type of estimation^34^. Given the controversies surrounding the interpretation of various indices of approximate fit in measurement invariance testing and considering our use of WLSMV to account for ordinal data, we decided to evaluate measurement invariance using the chi-square difference test provided in the MPlus shortcut option. We judged this method to be more reliable than use of approximate fit indices, particularly because our sample size was not overly large. Statistical significance in all analyses was determined by a p-value < 0.05.

### Ethics statement

The study was approved by the Swedish Ethical Review Authority (2022–03327-01). All participants were 15 years or older, and in accordance with Swedish ethical regulations, parental consent was not required. Participants provided informed consent on the initial survey question.

## Results

### Demographics

Mean and standard deviations of all scales for the two sports (Athletics and orienteering) as well as for self-assigned gender (female and male athletes) are shown in Table 2. Gender differences related to low self-stigma (SPSI) and anxiety (HADS) were revealed, with female athletes reporting lower self-stigma and higher anxiety scores than males. No other significant differences in the assessments across sports or gender were found. Table 2 also displays skewness, kurtosis, and McDonald’s omega (ω) for the scales. All scales demonstrated acceptable ω values (> 0.70) and were, except for the CART-Q, approximately normally distributed.

**Table 2:** Descriptive statistics of all scales for the two sports (Athletics and orienteering) and self-assigned gender. Skewness, kurtosis and McDonald’s omega (ω) for all scales are also displayed.

Scales	Mean sport		Mann-Whitney U-test: Athletics vs. orienteers			Mean gender		Mann-Whitney U-test: female vs. males			Mean Total sample (SD)	Skewness	Kurtosis	ω
	Athletics (SD)	Orienteering (SD)	Mann-Whitney U (z)	p	r	Females (SD)	Males (SD)	Mann-Whitney U (z)	p	r				
Psychological safety (TPSS; range 7–49)	39.37 (6.47) *n* = 233	39.76 (6.66) *n* = 137	15225.50 (−0.74)	0.46	− 0.04	38.99 (6.88) *n* = 197	40.11 (6.08) *n* = 173	15657.50 (−1.35)	0.18	− 0.07	39.52 (6.53) *n* = 370	− 0.79	0.51	0.72
Mentally healthy environment (SPSI; range 0–16)	11.61 (3.43) *n* = 233	12.04 (3.49) *n* = 136	14526.50 (−1.34)	0.18	− 0.07	11.64 (3.53) *n* = 196	11.92 (3.38) *n* = 173	16284.50 (−0.66)	0.51	− 0.03	11.77 (3.46) *n* = 369	− 0.61	− 0.18	0.88
Mental health literacy (SPSI; range 0–16)	10.20 (3.81) *n* = 233	10.87 (3.06) *n* = 136	14493.00 (−1.37)	0.17	− 0.07	10.12 (3.61) *n* = 197	10.82 (3.47) *n* = 172	14993.00 (−1.91)	0.06	− 0.10	10.45 (3.56) *n* = 369	− 0.35	− 0.39	0.85
Low self-stigma (SPSI; range 0–12)	7.63 (3.15) *n* = 233	8.20 (2.94) *n* = 136	14233.00 (−1.64)	0.10	0.08	8.19 (3.02) *n* = 196	7.44 (3.11) *n* = 173	14524.00 (−2.39)	0.02*	− 0.12	7.84 (3.08) *n* = 369	− 0.49	− 0.47	0.81
Anxiety (HADS; range: 0–21)	6.70 (3.73) *n* = 233	6.29 (3.85) *n* = 138	14944.50 (−1.14)	0.25	− 0.06	7.64 (3.84) *n* = 198	5.31 (3.30) *n* = 173	10993.50 (−5.97)	< 0.001***	− 0.31	6.55 (3.78) *n* = 371	0.51	− 0.07	0.79
Depression (HADS; range: 0–21)	3.88 (3.12) *n* = 233	3.94 (3.46) *n* = 138	15755.00 (−0.32)	0.74	− 0.02	4.24 (3.49) *n* = 198	3.50 (2.89) *n* = 173	15179.00 (−1.91)	0.06	− 0.10	3.90 (3.24) *n* = 371	1.13	0.74	0.80
Coach-athlete relationship (CART-Q; range: 10–70)	63.02 (7.72) *n* = 233	62.00 (8.72) *n* = 133	14488.00 (−1.04)	0.30	− 0.05	66.25 (8.52) *n* = 195	67.51 (9.98) *n* = 171	15568.50 (−1.10)	0.27	− 0.06	62.65 (8.10) *n* = 366	−1.93	4.57	0.90

### Construct validity

The strongest positive correlations between the psychological safety inventories and the validation instruments were observed for the TPSS and the SPSI subscale mentally healthy environment (Table 3). Although all psychological safety scales (TPSS, SPSI subscales) were significantly and negatively correlated with anxiety and depression scores, the TPSS and the subscale mentally healthy environment (SPSI) showed the strongest negative correlations.

**Table 3:** Associations between psychological safety inventories and validation instruments (Spearman rank-order correlations).

Scales	Psychological safety (TPSS)	Mentally healthy environment (SPSI)	Mental health literacy (SPSI)	Low self-stigma (SPSI)
Anxiety (HADS)	− 0.39** (*n* = 370	− 0.37** (*n* = 369)	− 0.22** (*n* = 369)	− 0.18** (*n* = 369
Depression (HADS)	− 0.35** (*n* = 370)	− 0.36** (*n* = 369)	− 0.26** (*n* = 369)	− 0.25** (*n* = 369)
Coach-athlete relationship (CART-Q)	0.39** (*n* = 366)	0.46** (*n* = 365)	0.23** (*n* = 364)	0.13* (*n* = 364)

*p <.05; **p <.001.

### Confirmatory factor analyses (CFA)

Because no significant differences were found between Athletics athletes and orienteers’ mean scores on the psychological safety inventories (Table 2), the study participants were analyzed as one sample in the CFA. Data screening with Mahalanobis distance identified 12 multivariate outliers (χ2(7) ≥ 24.32, *p* ≤.001) for the TPSS. One case had incomplete data and 13 cases were excluded from further analyses, resulting in a final sample of 358 cases (females: *n* = 192; males: *n* = 166) used in the measurement invariance analyses of TPSS. For SPSI, 8 cases were identified as multivariate outliers and four were identified with incomplete data. The final sample used for the SPSI included 359 cases (females: *n* = 192; males: *n* = 167).

Results from all CFAs are presented in Table 4. The CFA conducted on the TPSS with one latent factor indicated a good model fit across all fit indices, while the one latent factor model of the SPSI revealed a poor model fit. Analyses of the proposed SPSI three-factor correlated model and the higher order model, with the higher order factor specified to load on three latent factors, showed model fit to be acceptable (with CFI indicating an excellent fit and the RMSEA suggesting a mediocre model fit).

**Table 4:** Confirmatory analyses for á priori hypothesised models of TPSS and SPSI with chi-square (Χ²) and degrees of freedom (df). Model fit evaluated by comparative fit index (CFI), root mean square error of approximation (RMSEA) with 90% confidence interval (CI).

Á priori hypothesized models	Χ^2^	df	CFI	RMSEA (90% CI)
TPSS:				
First order model with one latent factor	35.67*	14	0.984	0.066 (0.039–0.093)
SPSI:				
First order model with one latent variable	1411.58*	44	0.770	0.294 (0.281–0.308)
First order model with three latent correlated latent factors	186.27*	41	0.976	0.099 (0.085–0.114)
Second order model with one higher order latent factor and three first order latent factors	186.27*	41	0.976	0.099 (0.085–0.114)
Both scales:				
TPSS first order model and SPSI first order model with three correlated latent variables	330.58*	129	0.970	0.066 (0.058–0.075)

*p >.005.

Figure 2 presents the post hoc analysis where the one latent factor solution of the TPSS and the three-factor measurement model of the SPSI were analyzed within the same model. Mahalanobis distance identified 13 multivariate outliers (χ2(18) ≥ 42.31, *p* >.001) and four cases had incomplete data. Analyses were performed on 354 cases (females: *n* = 188; males: *n* = 166). The one latent factor of the TPSS was specified to correlate with the three latent factors of the SPSI. As shown in Table 4, this combined model demonstrated an acceptable model fit with all fit indices reaching acceptable levels. The strongest relationship between the latent factors of TPSS and SPSI was found between psychological safety (TPSS) and mental healthy environment (SPSI), while the relationships between psychological safety (TPSS), mental health literacy (SPSI) and low-self stigma (SPSI) were weaker.

### Measurement invariance across gender

When females and males were analyzed separately, the TPSS (the first order model with one latent factor) displayed an acceptable to mediocre model fit for both genders (females: χ^2^ = 32.36(14), *p* <.001, CFI = 0.98, RMSEA = 0.08; males: χ^2^ = 30.11(14), *p* <.001, CFI = 0.98, RMSEA = 0.08). The SPSI (the first order model with three correlated latent factors) displayed an acceptable to poor model fit (females: χ^2^ = 138.74(41), *p* <.001, CFI = 0.97, RMSEA = 0.11; males: χ^2^ = 110.23(41), *p* <.001, CFI = 0.97, RMSEA = 0.10).

Measurement invariance tests were performed for the TPSS and SPSI respectively (Table 5). The shortcut chi-square test for difference testing suggested the TPSS to be metric and scalar invariant across genders. For the SPSI, the shortcut chi-square test for difference testing suggested the model to be metric but not scalar invariant across genders. To explore invariance of individual thresholds they were constrained on by one. The scalar-metric comparisons showed all single thresholds tested to be significant (*p* <.001) indicating them to be non-invariant.

**Table 5:** Measurement invariance (configural, metric and scalar) of the TPSS and the SPSI across genders. Configural invariance denotes the model fit achieved with only the factorial structure constrained across the groups of females and males, also serving as the baseline. Metric invariance is measured by constraining the factor loadings across gender. Scalar invariance requires the item thresholds to be identical for both genders.

Scale	Model fit		χ^2^ difference testing*		
	χ^2^	df	Value	df	p
TPSS (females *n* = 192; males *n* = 166):					
Configural invariance	61.84*	28	--	--	--
Metric invariance	54.83*	34	4.83	6	0.56
Scalar invariance	86.95*	66	36.47	32	0.27
SPSI (females *n* = 192; males *n* = 167):					
Configural invariance	249.95*	82	--	--	--
Metric invariance	249.56*	90	8.12	8	0.42
Scalar invariance	323.65*	119	89.52*	29	< 0.001

* Chi-square difference testing was performed by use of MPlus shortcut option which automatically performs scalar corrections for WLSMV. Criteria for non-invariance *p* <.05.

## Discussion

This validation study of instruments measuring psychological safety confirmed the internal consistency of the investigated scales and their proposed factor structures: a one-factor solution for the TPSS and a three-factor correlated solution for the SPSI. Consistent with the findings of Rice et al.^15^, a one-factor solution for the SPSI was not supported and a higher order model was not found superior to the three-factor correlated solution. The TPSS was found to be fully invariant across genders, while scalar invariance was not supported for the SPSI. Indications of non-invariance across gender present a significant challenge for researchers aiming to conduct gender comparisons with the scale. When invariance is questionable, any observed score differences may reflect measurement bias rather than true differences in the construct, rendering such comparisons scientifically meaningless^32,40^. Further research is desirable to investigate the measurement invariance of the scales across genders, sports, cultures and other groups that may be of interest for comparisons. Our results, however, suggest that if researchers are faced with the choice between the scales for studying gender differences related to psychological safety in sports, the TPSS may be preferable to the SPSI.

Regarding construct validity, the TPSS correlated with the indicators of mental health and the quality of the coach-athlete relationship in the theoretically expected direction. The mentally healthy environment subscale of the SPSI exhibited a similar pattern as the TPSS. Overall, the moderate strength of the correlation between the TPSS and the SPSI mentally healthy environment subscale when the two scales were jointly analyzed in the post hoc CFA suggests that these two scales partly, but not entirely, target a similar concept. The other two subscales, mental health literacy and low self-stigma, exhibited a divergent pattern suggesting that they measure constructs that are conceptually distinct from both the TPSS and the mentally healthy environment subscale. These findings are important, yet anticipated, given the semantic differences in the definition of psychological safety across organizational and sports contexts^2,3,14,15^. Psychological safety has been extensively investigated in organizational settings, with several theoretical perspectives proposed to explain its mechanisms at different levels (individual, team, or organizational) and its influence on work outcomes^3^. In comparison, an aim of introducing the concept in sports appears to have been the identification of predictors of future mental health, as reflected in both the definition proposed by the IOC and the SPSI developed from this perspective^14,15^. However, the specific purpose of the application of the psychological safety concept in sports remains unclear, which also is noticed in that the transfer of the organizational meaning to sports settings has been contested^6^. The IOC publication^14^ that presents the definition of psychological safety which the SPSI builds upon offers limited guidance because references to empirical scientific studies are lacking. This raises the question of whether describing the SPSI as a ‘sport psychological safety scale’ is constructive. Despite an acceptable model fit and internal consistency, the SPSI seems to lack a clear, empirically supported definition or theoretical foundation to guide researchers’ interpretation of scores obtained with the scale. In other words, it is unclear what the scale truly measures.

The empirical knowledge on how psychological safety in sports is perceived and influenced by various factors, as well as its relationship to different outcomes (e.g., performance, health, long-term development, motivation) is currently limited. The diverse and vague descriptions pose a risk of constraining scientific progress and practical assessments of psychological safety in sports^5,6^. Experiences from outside the sports domain suggest that researchers need to study not only benefits but also potential drawbacks in various settings related to psychological safety^3^. It is essential to ensure that recommendations related to psychological safety in sports are founded on empirical studies with high methodological quality including valid assessments. This implies that continued research is warranted on what the SPSI measures by comparing its subscales to existing scales, such as those for mental health literacy^41,42^ and stigma^43,44^. The domain (i.e., the target concept, attribute, unobserved behavior, etc.) should be clearly articulated and defined. A well-defined, theoretically supported domain is crucial for establishment of construct validity and the boundaries of the construct that the scale should assess^45^. Moreover, the existing literature should be reviewed to establish whether present instruments could serve the same purpose as the intended new scale. If similar scales exist, a justification for developing a new scale is required, along with an explanation of how it differs from existing instruments^45^. Finally, when adopting the TPSS and SPSI in sports, researchers should be cautious of the jingle fallacy, which occurs when two different scales are assumed to assess the same construct because they share the same name, but in fact, assess different constructs^46^. Jingle fallacies can lead to confusion and misinterpretation, making it challenging to compare and integrate findings across studies. When transferring a concept from one setting to another, which applies to the TPSS and SPSI in the sports setting, researchers should also be actively aware of the risk of concept creep, which can distort the original meaning of the term through semantic shifts and subsequently undermine the scientific and practical value of the construct^47^.

This study offers new insights into the psychometric properties of two scales used to measure psychological safety in sports. However, some limitations should be noted when interpreting the results. Despite the sample compromised elite athletes across a range of ages, from junior elite to senior elite levels, it was predominantly composed of young developing athletes. Additionally, the study included only individual sports athletes, specifically Athletics athletes and orienteers. It is possible that psychological safety, when assessed according to its organizational meaning, is a more significant construct for use with athletes participating in team sports than individual sports. This hypothesis could not be tested in this study. The population studied was from a single Scandinavian country, and the cultural and educational background may also influence the results. In addition, the study did not include any coaches, support staff or other groups involved in sports environments. Therefore, future research should include both individual and team sports, as well as coaches and staff from various countries and sporting levels, to further evaluate the psychometric properties of the scales.

In conclusion, the results of this study underscore that psychological assessments used in sports should be based on judiciously developed operational definitions and carefully validated. The TPSS exhibited acceptable psychometric properties for assessing psychological safety in an elite sports context. While the SPSI three-factor correlated model demonstrated a robust factor structure and internal consistency, it was not invariant across genders. Concerns about its construct validity were also raised. These findings underscore a need for caution when using the SPSI as a measure of psychological safety in sports settings.

## Data Availability

Data are available from the corresponding author on reasonable request.
